# State of the art and future perspectives on the management of spheno-orbital meningiomas: a meta-analysis comparing transorbital and transcranial approaches

**DOI:** 10.3389/fonc.2026.1773654

**Published:** 2026-05-12

**Authors:** Edoardo Porto, Giorgio Fiore, Arianna Barbotti, Giulio A Bertani, Massimiliano Del Bene, Giovanni Marfia, Francesco Corrivetti, Matteo de Notaris, Domenico Solari, Gustavo Pradilla, Hani J Marcus, Marco Locatelli, Francesco DiMeco

**Affiliations:** 1 Department of Neurosurgery, Fondazione IRCCS Istituto Neurologico Carlo Besta, Milan, Italy; 2 Department of Neurosurgery, Emory University, Atlanta, GA, United States; 3 Unit of Neurosurgery, Fondazione IRCCS Ca’ Granda Ospedale Maggiore Policlinico, Milan, Italy; 4 UCL Queen Square Institute of Neurology, University College London, London, United Kingdom; 5 Laboratory of Neuroanatomy, European Biomedical Research Institute of Salerno (EBRIS) Foundation, Salerno, Italy; 6 Neurosurgery Unit, University Hospital “San Giovanni di Dio e Ruggi D’Aragona”, Salerno, Italy; 7 Division of Neurosurgery, Department of Neurosciences, Reproductive and Odontostomatological Sciences, Universita’ degli Studi di Napoli Federico II, Naples, Italy; 8 Victor Horsley Department of Neurosurgery, National Hospital for Neurology and Neurosurgery, London, United Kingdom; 9 Department of Oncology and Hematology-Oncology, Università degli Studi di Milano, Milan, Italy; 10 Department of Neurosurgery, Johns Hopkins University, Baltimore, MD, United States

**Keywords:** endoscopic transorbital approach, meningioma, skull base surgery, spheno-orbital meningioma, transcranial surgery

## Abstract

**Background:**

Spheno-orbital meningiomas (SOMs) are challenging skull base tumors requiring a balance between oncologic control and functional–aesthetic outcomes. Endoscopic transorbital approaches (ETOA) have recently gained popularity as minimally invasive alternatives to standard transcranial techniques, but comparative evidence is lacking.

**Methods:**

A systematic review and meta-analysis were conducted following PRISMA guidelines. PubMed/MEDLINE, Embase, and Scopus were searched from inception to the final search date. Studies reporting outcomes after ETOA or standard transcranial approaches were included. Primary outcomes included improvement in proptosis and visual function, extent of resection, and complications. Secondary outcomes included tumor progression and recurrence. Random-effects generalized linear mixed models were used to pool proportions. Mixed-effects meta-regression evaluated the association between approach and outcomes.

**Results:**

Fifty-three studies encompassing 2016 patients were included. A total of 155 (7.7%) ETOA and 1864 (92.3%) transcranial approaches were performed. Proptosis improvement was greater with ETOA (96% vs 72%) while visual improvement was similar (ETOA 31% vs standard 33%; p=0.857). Gross total resection (GTR) was lower with ETOA (21.1% vs 48.0%) and favored transcranial approaches after sensitivity analysis excluding influential studies (OR 5.00, p=0.047). Complication rates did not differ significantly between approaches, with a numerically lower rate observed after ETOA compared with standard transcranial surgery (18% vs 26%; p = 0.165). Median follow-up was shorter in the ETOA series (18 vs 52 months); recurrence and progression were lower after ETOA but likely influenced by follow-up duration.

**Conclusions:**

ETOA demonstrates superior proptosis improvement compared with standard transcranial approaches, comparable visual outcomes, and lower rates of GTR. Complication rates did not differ significantly. In appropriately selected SOMs, ETOA represents a valid surgical strategy within a tailored, goal-oriented approach, while standard transcranial techniques remain a workhorse for cases requiring extensive intracranial or dural control. Longer follow-up is required to clarify long-term tumor control.

## Introduction

Spheno-orbital meningiomas (SOM) are uncommon skull base tumors characterized by a dual intraorbital and intracranial extension, arising from the sphenoid wings. Their insidious growth pattern often leads to delayed diagnosis and complex clinical presentations, with progressive hyperostosis, optic apparatus compression, and disfiguring proptosis (1). Achieving optimal management remains challenging, as SOMs frequently envelop neurovascular structures and invade the orbit, making radical resection difficult and recurrence relatively common. Historically, treatment strategies have been dominated by various transcranial approaches that prioritize maximal safe tumor and bone removal while preserving vision and ocular function (1–5).

Standard transcranial routes, such as the frontotemporal (pterional), orbitozygomatic, and extended skull base approaches, provide wide exposure and are the most adopted surgical techniques. These approaches facilitate comprehensive resection of the tumor and hyperostotic bone but carry notable morbidity related to brain retraction, cosmetic sequelae, and manipulation of critical neurovascular structures (6). Despite refinements in microsurgical technique and intraoperative navigation, the balance between oncologic control and functional–aesthetic outcomes remains a topic of debate, especially in cases with predominant orbital involvement. As a result, surgeons have increasingly explored less invasive strategies that maintain therapeutic effectiveness while reducing collateral morbidity (1, 6, 7).

**Figure 1:**
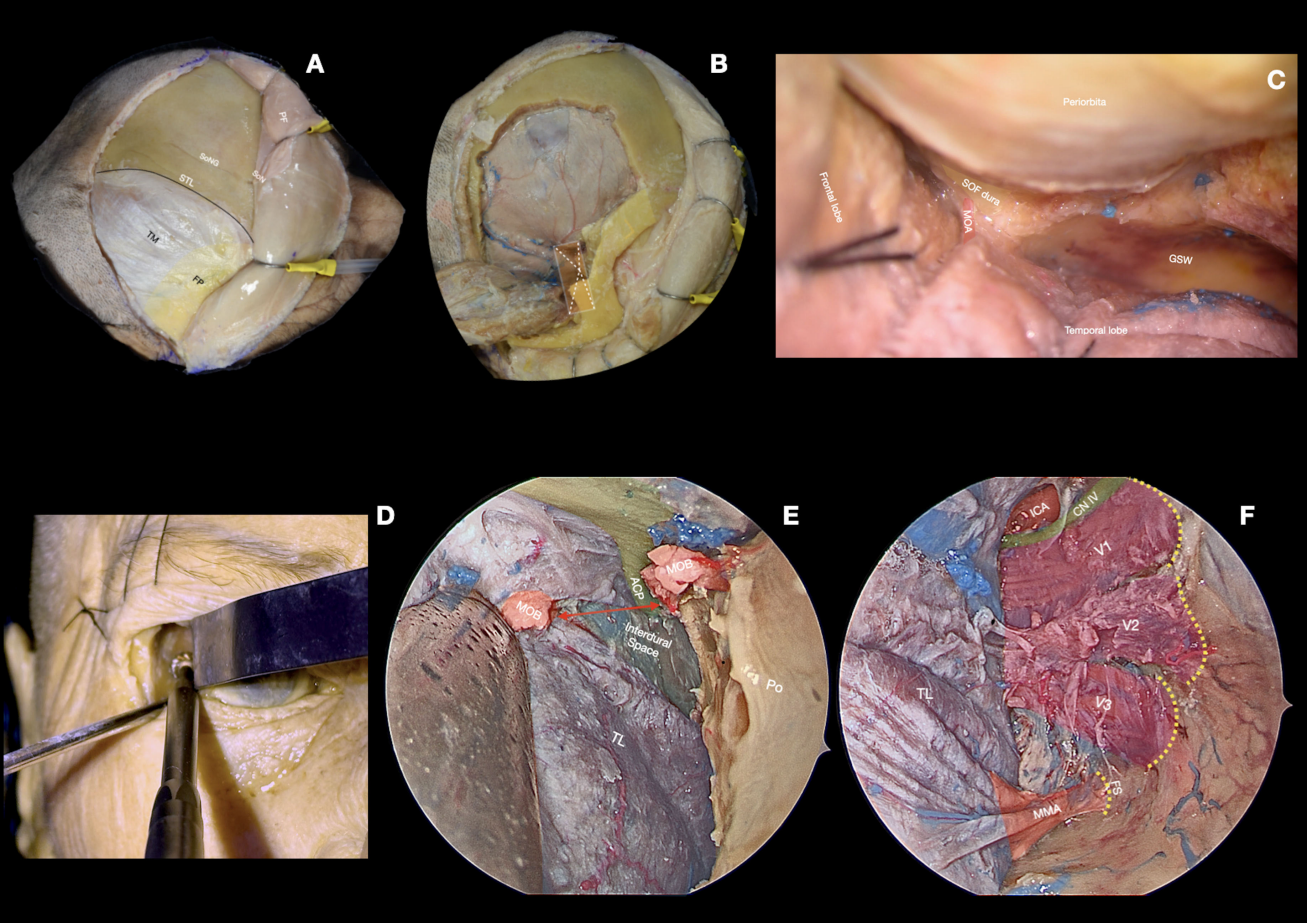
Cadaveric dissections illustrating surgical corridors for SOMs. **(A–C)** show a standard frontotemporal transcranial approach, highlighting exposure of the middle cranial fossa and adjacent neurovascular structures. **(D–F)** depict an endoscopic transorbital approach (ETOA) directed toward the middle cranial fossa. ACP, anterior clinoid process; CN IV, fourth cranial nerve; FP, fat pad; FS, foramen spinosum; GSW, greater sphenoid wing; ICA, internal carotid artery; MMA, middle meningeal artery; MOB, meningo-orbital band; Po, periorbita; SOF dura, superior orbital fissure dura; SoNG, supraorbital nerve groove; STL, superior temporal line; TL, temporal lobe; TM, temporalis muscle; V1, first trigeminal branch; V2, second trigeminal branch; V3, third trigeminal branch.

In the past decade, there has been a marked surge in the use of transorbital approaches, fuelled by anatomical understanding of the orbit, advances in endoscopy, and minimally invasive skull base techniques (8, 9). Endoscopic transorbital approaches (ETOA), particularly the superior eyelid transorbital approach, offer direct (10), cosmetically favorable access to the sphenoid wings, lateral orbit, and middle cranial fossa without the need for large craniotomies (11–14). Early reports suggest potential benefits, including reduced surgical footprint, shorter hospitalization, minimized soft-tissue disruption, and improved aesthetic outcomes. As experience accumulates, these techniques are increasingly proposed as viable alternatives or adjuncts to conventional transcranial approaches, especially in cases with limited intradural extension and predominant lateral orbital disease (11, 15–19) (**Figure 1**).

Despite growing enthusiasm, evidence comparing ETOA and traditional transcranial approaches remains fragmented and heterogeneous. Variation in patient selection, extent of resection, reconstruction strategies, and definitions of outcome complicate attempts to draw definitive conclusions regarding safety and efficacy of such techniques. A comprehensive synthesis of the current literature is therefore necessary to determine the relative advantages, limitations, and appropriate indications of each approach. This meta-analysis aims to quantitively evaluate existing studies describing either ETOA or standard transcranial techniques in the management of SOMs, with particular attention to surgical and clinical outcomes, tumor control, and perioperative morbidity.

## Materials and methods

### Study design and eligibility criteria

This study was conducted as a systematic review and meta-analysis in accordance with the PRISMA guidelines (20). The objective was to compare surgical outcomes of SOMs treated with standard transcranial approaches or ETOA.

Eligible studies met the following criteria:

1. included adult patients with SOMs;
2. reported outcomes following either transcranial or endoscopic transorbital resection;
3. provided extractable data for at least one predefined outcome of interest;
4. were original clinical studies (randomised or non-randomised), including retrospective and prospective cohort studies.

Case reports, review articles, editorials, technical notes without outcome data, conference abstracts, and non-English-language studies were excluded. When overlapping patient cohorts were identified, the most recent or most comprehensive study was retained.

### Literature search and study selection

A systematic literature search was performed in PubMed/MEDLINE, Embase, and Scopus from database inception to the date of the final search. The search strategy combined controlled vocabulary and free-text terms related to SOMs and surgical approach, including combinations of “spheno-orbital meningioma”, “sphenoid wing meningioma”, “transorbital”, “endoscopic”, “microsurgical”, “orbit”, “meningioma”, and “skull base”.

All retrieved records were imported into a reference management software, and duplicate entries were removed. Titles and abstracts were independently screened by two reviewers (EP and GF) to identify potentially eligible studies. Full texts of selected articles were then reviewed for eligibility. Discrepancies were resolved by consensus.

### Data extraction

Two authors (GF and EP) independently screened titles, abstracts, and full texts for inclusion. Any discrepancies were resolved by consensus. Data extraction was performed independently by the same reviewers using a predefined data collection template. Extracted variables included study characteristics (first author, year of publication, and number of patients), surgical approach (standard transcranial approaches or endoscopic transorbital approaches), follow-up duration, and reported surgical outcomes.

### Outcomes

Primary outcomes reflected both effectiveness and safety and included proptosis and visual function improvement, extent of resection, and complications. Proptosis improvement was defined as a postoperative reduction in proptosis based on clinical assessment, according to study-specific criteria. Visual improvement was defined as any reported postoperative improvement in visual acuity or visual field deficits compared with the preoperative status. The extent of resection was evaluated as two separate binary outcomes: gross total resection (GTR) and subtotal resection (STR), as reported in the original studies. Complications were defined as any postoperative adverse event attributed to the surgical procedure, including cranial nerve deficits, cerebrospinal fluid leak, infection, vascular injury, or other surgery-related morbidity.

Secondary outcomes included tumor progression and recurrence. For tumor control analyses, follow-up duration (in months) was incorporated into the statistical models to account for differences in time at risk across studies.

Variables reported in **Table 1** but not included among the predefined outcomes (e.g., proptosis impairment, visual impairment, and near-total resection [NTR]) were extracted as descriptive measures; proptosis and visual impairment were defined as lack of postoperative improvement, while NTR was defined according to study-specific criteria as resection leaving minimal residual tumor between GTR and STR.

**Table 1:** General data of the studies included.

General features					Outcomes																
Author	Year	Cases	ETOA %	Standard approach %	Proptosis improvement ETOA %	Proptosis impairment standard %	Visual impairment ETOA %	Visual improvement standard %	GTR ETOA %	GTR standard %	NTR ETOA %	NTR standard %	STR ETOA %	STR standard %	Complications ETOA %	Complications standard %	Recurrence ETOA %	Recurrence standard%	Progression ETOA %	Progression standard %	Follow-up (months)
Agi et al. (22)	2020	20		100		90		55		55				45		40		0		25	57.3
Almeida et al. (23)	2018	2	100		100		100		0		0		100		50		0		0		6.0
Bikmaz et al. (24)	2007	67		100		100		70.15		20.9				79.1		5.97		1.49			36.0
Boari et al. (25)	2013	40		100		92.5		27.5		57.5				45		17.5		10			72.0
Bowers et al. (26)	2016	33		100		51.52		45.45		93.94				6.06		15.15		6.06			54.0
Cannon et al. (27)	2009	12		100		50		16.67		0				100		33.33		0		33.33	31.0
Colombo et al. (28)	2022	3	100		100		33.33		0		0		100		0		0		0		12.0
Dallan et al. (29)	2025	52	100		96.15		30.77		28.85		23.08		48.08		21.15		11.54				45.0
Dalle Ore et al. (30)	2020	54		100				22.22		38.89		51.85		9.26		44.44		22.22		22.22	30.0
De Jesus et al.	2001	6		100		83.33		50		83.33				16.67		0		0		16.67	48.0
Di Somma et al. (17)	2023	7	100		100		28.57		28.57		0		71.43		0		0		0		22.0
Forster et al. (33)	2014	18		100		100		44.44		72.22				27.78		27.78		27.78		11.11	43.0
Freeman et al. (34)	2017	25		100		76		16		56				44		32		8		40	45.0
Gomes dos Santos et al.	2022	40		100		0		0		5		60		35		25		27.5			39.0
Goncalves et al. (35)	2020	21	100	0	100		42.86		0		0		100		4.76		0		0		6.0
Gonen et al. (36)	2017	27		100		77.78		29.63		51.85				48.15		29.63		14.81		7.41	41.0
Heufelder et al. (37)	2009	21		100		47.62		9.52		100						0		42.86			65.0
Honig et al. (38)	2010	30		100				50		33.33				66.67				10		20	33.7
In Woo et al. (39)	2021	18	100		94.44		44.44		16.67				83.33		33.33		0		11.11		20.4
Kim et al. (40)	2022	33		100		57.58		9.09		0		27.27		72.73		15.15		21.21			18.0
Kong et al.	2020	41	100		48.78		4.88		51.22				48.78		14.63		2.44		4.88		15.9
Leroy et al. (41)	2016	70		100		68.57		14.29		31.43				68.57		61.43		28.57			60.0
Li et al. (42)	2009	37		100		100		48.65		24.32				75.68		24.32		18.92		13.51	36.0
Liu et al. (43)	2024	100		100		85		48		34				66		12		3		14	52.3
Luetjens et al. (44)	2011	3		100		100		66.66		66.67				33.33		33.33		33.33			30.0
Marcus et al. (45)	2013	19		100		52.63		57.89		57.89				42.11		26.32		0		0	60.0
Mariniello et al. (46)	2024	80		100		67.5		30		65				35				15		22.5	136.0
Masalha et al. (47)	2021	69		100						37.68		56.52		0		14.49		30.43			121.0
Menon et al. (48)	2020	17		100		52.94		23.53	0	11.76		0		88.24		76.47				88.24	36.0
Mirone et al. (49)	2009	71		100		74.65		42.25		83.1				16.9		26.76		4.23		4.23	77.0
Moscovici et al. (50)	2024	45		100		73.33		44.44		48.89				51.11		4.44		4.44		15.56	76.0
Nagahama et al. (51)	2019	13		100		76.92		7.69		23.08				76.92		46.15		0		30.77	74.4
Oya et al. (3)	2011	39		100		64.1		35.9		38.46		51.28		10.26		28.21		17.95			41.0
Pace et al. (52)	2019	20		100		20		45		75				25		15		20			45.0
Park et al. (12)	2020	24	45.83	54.17					37.5	45.83			8.33	8.33	12.5	8.33	0	0	0	0	20.0
Planty-Bonjour et al. (53)	2024	22		100		95.45		36.36		31.82				68.18		13.64		0		27.27	65.0
Porto et al. (54)	2025	89		100		46.07		13.48		53.93		8.99		37.08		12.36		22.47			78.0
Ringel et al. (55)	2006	63		100		74.6		61.9		23.81				76.19		49.21				30.16	53.0
Roser et al. (56)	2005	82		100		63.41		13.41		37.8				62.2		21.95		10.98		19.51	62.2
Saeed et al. (6)	2011	66		100		25.76		60.61				100				33.33		16.67			102.0
Samadian et al. (57)	2020	57		100		100		12.28		100		0		0		29.82		12.28		10.53	46.0
Sandalcioglu et al. (58)	2005	16		100		0		0		68.75				31.25		68.75		56.25		50	68.0
Scarone et al. (59)	2009	30		100		90						100				60		10			61.0
Schick et al. (60)	2006	67		100		38.81		17.91		59.7				40.3		20.9		10.45			30.0
Shrivastava et al. (7)	2005	25		100		96		64		72				28		68		8		0	60.0
Simans et al. (61)	2013	18		100		88.89				38.89				61.11		33.33		27.78			54.0
Solmaz et al. (62)	2014	13		100		61.54		46.15		30.77				69.23		0		0		0	26.0
Talacchi et al. (63)	2014	47		100		8.51				51.06				48.94		19.15		29.79		4.26	52.0
Terrier et al. (64)	2018	90		100		84.44		43.33		32.22				67.78		18.89		4.44		26.67	65.0
Young et al. (65)	2019	24		100		20.83		50								41.67				33.33	87.0
Zamanipoor Najafabadi et al. (66)	2021	19		100		21.05		100		78.95		0		21.05		63.16		10.53			28.0
Zohdy et al. (67)	2024	66		100		84.85		45.45		12.12				87.88							6.0
Zoli et al. (68)	2023	45		100		68.89		26.67		62.22				37.78		24.44		0		15.56	83.4

### Statistical analysis

For each outcome, pooled event rates were estimated using random-effects meta-analysis of proportions, fitted with binomial generalized linear mixed models (GLMMs) with logit link and maximum likelihood estimation. Outcomes were modelled as the number of events over the total number of patients per study (e.g., STR/n, GTR/n, Visual Improvement/n, Proptosis improvement/n, Complications/n). Pooled proportions and 95% confidence intervals (CI) were obtained by back-transformation of model estimates using the inverse logit function. Between-study heterogeneity was quantified using τ² and I² statistics.

To assess the influence of surgical approach, mixed-effects meta-regression models were fitted with surgical approach as a study-level moderator. The significance of the moderator was assessed using the QM statistic. The approach regression coefficient represents the difference in log-odds between approaches. Odds ratios (ORs) with 95% CIs were derived by exponentiation of model coefficients. Meta-regression analyses for tumor control outcomes were not performed when substantial differences in follow-up duration were present between approaches, to avoid time-at-risk bias.

To evaluate the robustness of pooled estimates and the stability of meta-regression results, a structured influence analysis was conducted for each outcome and for both surgical approaches.

For models fitted using binomial random-effects GLMMs, influence diagnostics were computed manually using a leave-one-out (LOO) procedure. Each study was sequentially excluded, and the GLMM was refitted. For each iteration, changes in the regression coefficient for the surgical approach moderator (ETOA vs standard) were recorded. Influence was quantified using two complementary metrics:

1. the change in the estimated regression coefficient (beta difference), and
2. the corresponding change in the predicted pooled proportion for the outcome of interest.

Studies associated with the largest shifts in the regression coefficient or predicted proportions were classified as influential. Studies showing intermediate but appreciable shifts were classified as moderately influential. These classifications were used to inform predefined sensitivity analyses.

Sensitivity analyses were subsequently performed by refitting each model after exclusion of influential studies. For each sensitivity model, pooled proportions, between-study heterogeneity (τ² and I²), and the effect of surgical approach were recalculated using meta-regression. Odds ratios were obtained by exponentiating log-odds coefficients, and 95% confidence intervals were derived accordingly. Sensitivity analyses were used to assess whether the direction and magnitude of the association between surgical approach and each outcome remained consistent after removal of influential studies.

All statistical analyses were conducted in R software 4.2.2 (R Foundation for Statistical Computing, Vienna, Austria; https://www.rproject.org/index.html [accessed on 31 May 2025]). Statistical significance was set at p < 0.05.

### Risk of bias

Risk of bias was assessed independently by two reviewers (EP and GF). The Joanna Briggs Institute (JBI) checklist for case series and cohort studies was used (21). Studies scoring ≥7 were considered methodologically acceptable. Discrepancies were resolved through consensus.

## Results

### Study selection and features

**Figure 2:**
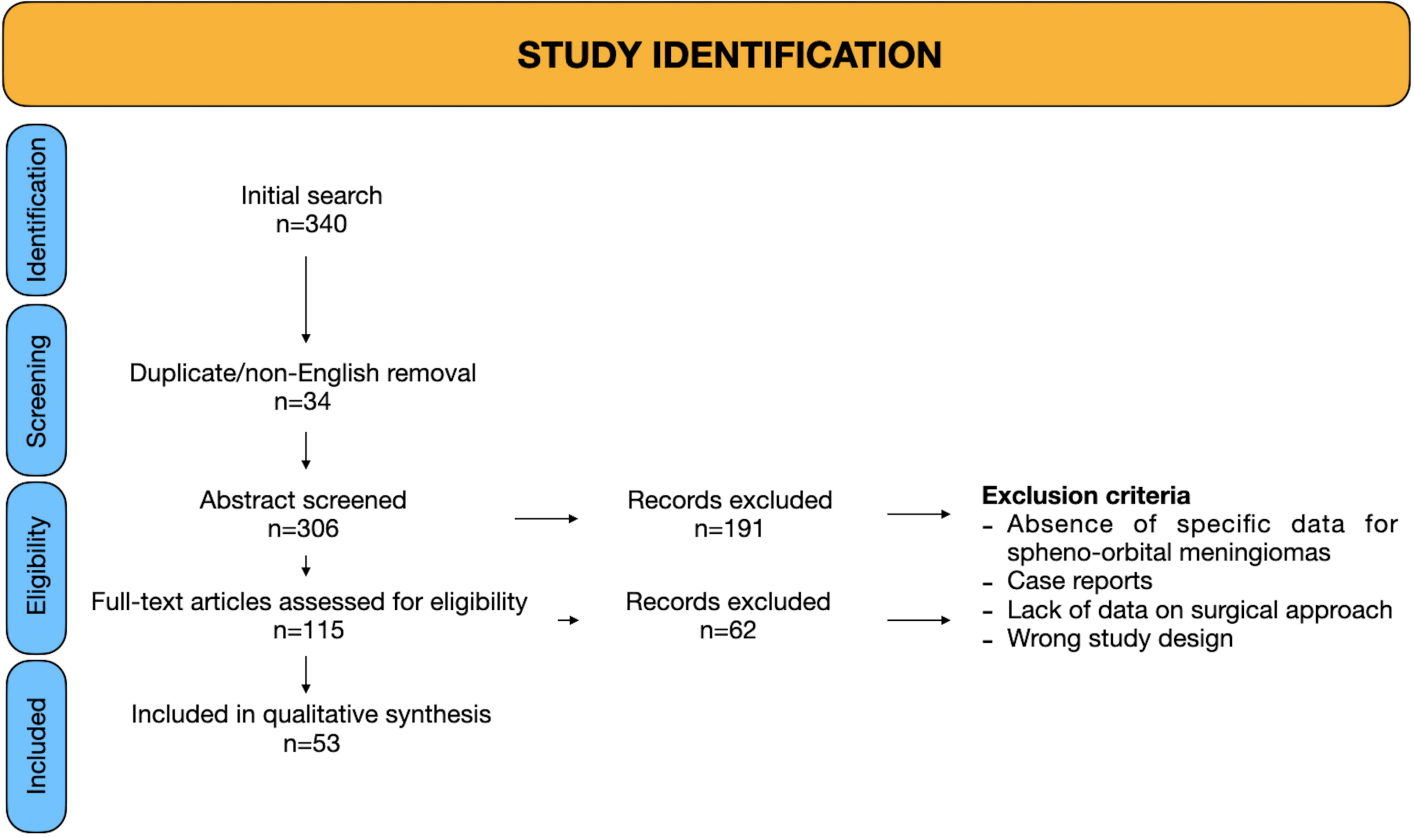
PRISMA chart.

A total of 53 studies were included in this meta-analysis (3, 6, 7, 11, 12, 17, 22–68), encompassing 2016 patients who underwent surgeries for SOM. The studies spanned publication years from 2001 to 2025, with cohort sizes ranging from 2 to 100 patients. The PRISMA flow diagram is provided in **Figure 2**.

Surgical approaches included ETOA (7.7%, n = 155) and the standard transcranial approach (92.3%, n = 1864). One study reported three cases of bilateral SOM in which bilateral surgical intervention was performed (44). General data of the studies included are presented in **Table 1**.

Descriptive variables such as proptosis impairment, visual impairment, and near-total resection (NTR) were inconsistently reported across studies and were therefore not included in the pooled analyses, but are presented in **Table 1** for completeness.

### Proptosis

The pooled proptosis improvement rate was 72% (95% CI 58–82%) following transcranial approaches and 96% (95% CI 75–100%) following ETOA, with substantial between-study heterogeneity.

**Figure 3:**
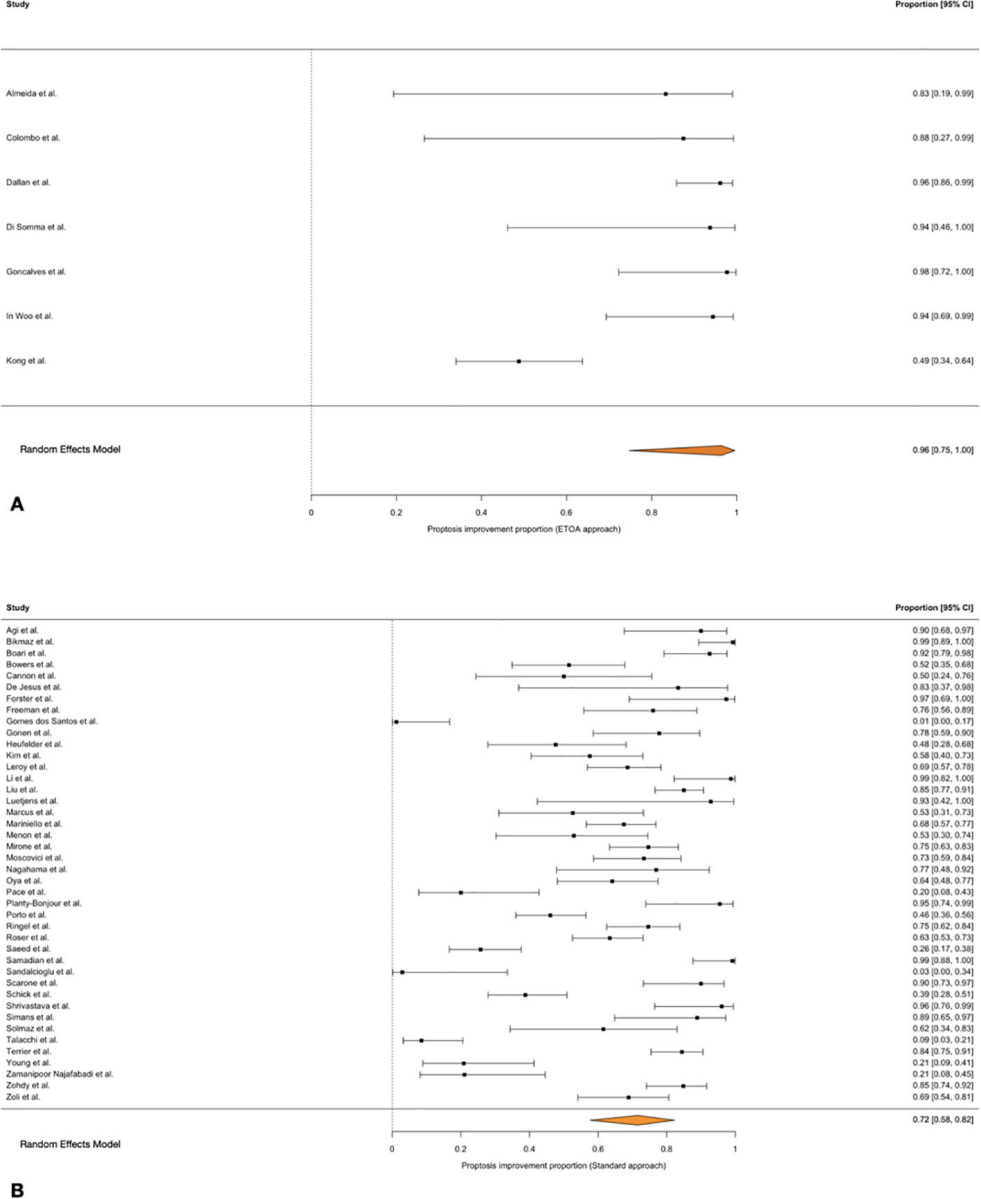
Forest plot showing the proptosis improvement rate across studies included. **(A)** ETOA; **(B)** Transcranial approach.

Meta-regression identified a significant association between surgical approach and proptosis improvement (QM = 5.49, p = 0.019). When expressed as ETOA versus transcranial approaches, ETOA was associated with significantly higher odds of proptosis improvement (OR = 11.0, 95% CI 1.48–83.3) (11) (**Figures 3A, B**).

### Visual outcome

The pooled visual improvement rate was 33% (95% CI 26–42%) following transcranial approaches and 31% (95% CI 16–51%) following ETOA, with substantial between-study heterogeneity.

**Figure 4:**
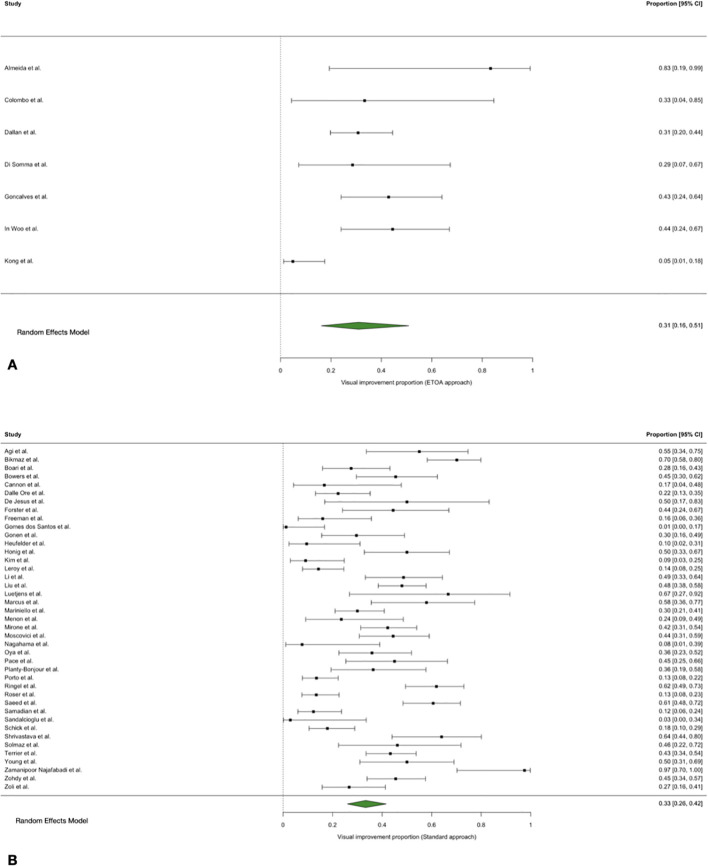
Forest plot showing the visual improvement rate across studies included. **(A)** ETOA; **(B)** Transcranial approach.

Meta-regression did not identify an association between surgical approach and visual improvement (QM = 0.03, p = 0.857), with comparable odds of improvement between approaches (OR = 1.10, 95% CI 0.40–3.01, transcranial approaches vs ETOA. Results were unchanged in sensitivity analysis excluding the most influential study (QM = 0.37, p = 0.543) (11) (**Figures 4A, B**).

### Subtotal resection

The pooled STR proportion for trasncranial approaches was 42.8% (95% CI 32.2–54.2%; I² = 93.7%; τ² = 2.01). For ETOA, the pooled STR proportion was 76.8% (95% CI 43.1–93.5%; I² = 89.3%; τ² = 2.83).

Meta-regression demonstrated a significant association between approach and STR (QM = 4.44, p = 0.035). ETOA was associated with higher odds of STR (OR = 4.00, 95% CI 1.10–14.52).

**Figure 5:**
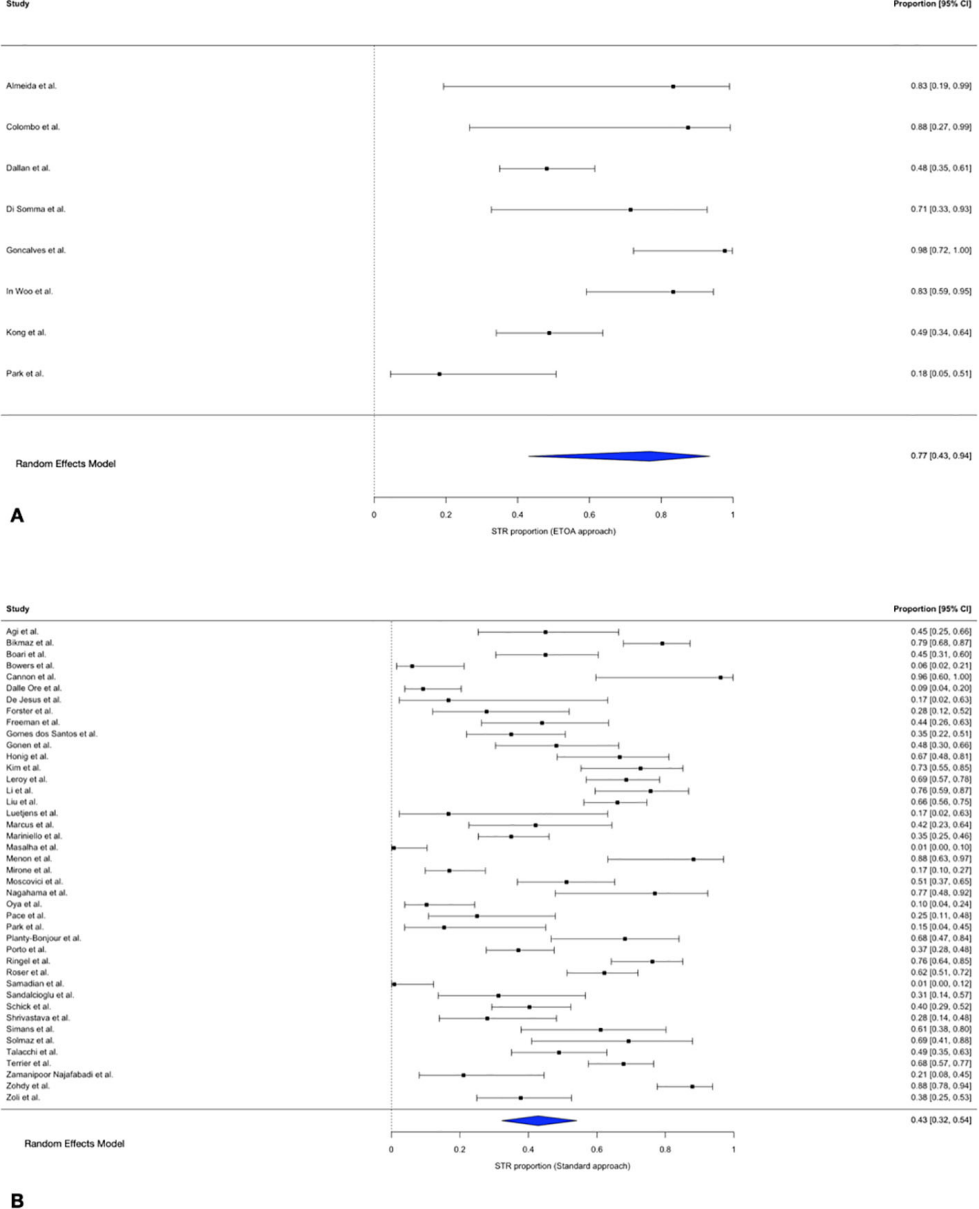
Forest plot showing the STR rate across studies included. **(A)** ETOA; **(B)** Transcranial approach.

In sensitivity analysis excluding influential studies (11, 12), the association strengthened (QM = 5.56, p = 0.018). Compared with ETOA, transcranial approaches had substantially lower odds of STR (OR = 0.094, 95% CI 0.013–0.671) (**Figures 5A, B**).

### Gross total resection

The pooled GTR proportion was 48.0% (95% CI 37.0–59.2%; I² = 93.9%; τ² = 2.03) for trasncranial approaches and 21.1% (95% CI 6.2–51.8%; I² = 87.4%; τ² = 2.49) for ETOA.

In the meta-regression analysis, surgical approach showed a non-significant trend towards an association with GTR (QM = 3.27, p = 0.071), with higher odds of GTR for transcranial approaches compared with ETOA (OR = 3.28, 95% CI 0.90–11.86).

**Figure 6:**
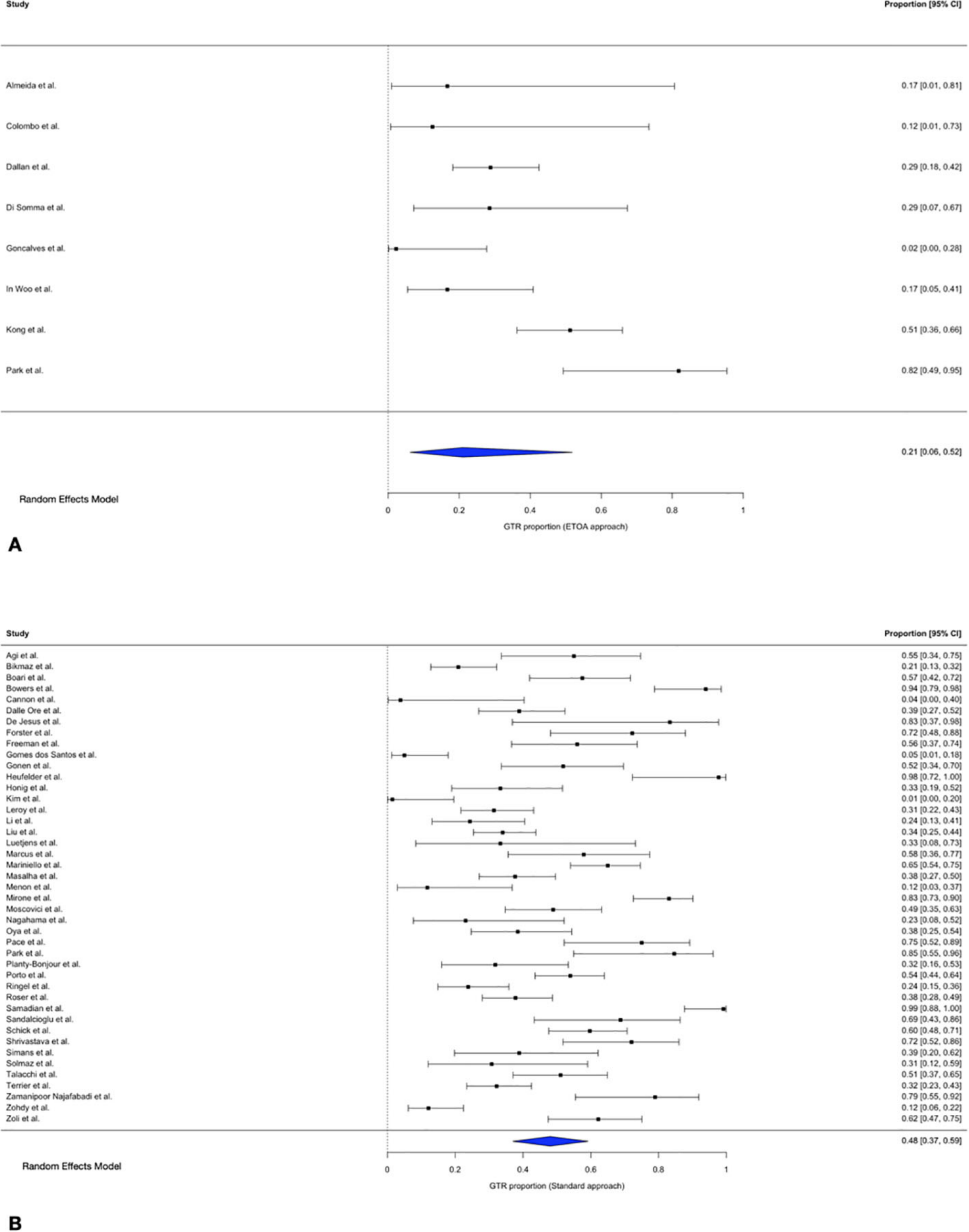
Forest plot showing the GTR rate across studies included. **(A)** ETOA; **(B)** Transcranial approach.

After exclusion of influential studies (11, 12), the association became statistically significant (QM = 3.96, p = 0.047). In this sensitivity analysis, transcranial approaches were associated with significantly higher odds of GTR compared with ETOA (OR = 5.00, 95% CI 1.02–25.0), corresponding to an approximately fivefold increase in the odds of achieving GTR (**Figures 6A, B**).

### Complications

The pooled complication rate was 26% (95% CI 20–32%) for trasncranial approaches and 18% (95% CI 12–26%) for ETOA, with substantial between-study heterogeneity (I² = 81.4%).

**Figure 7:**
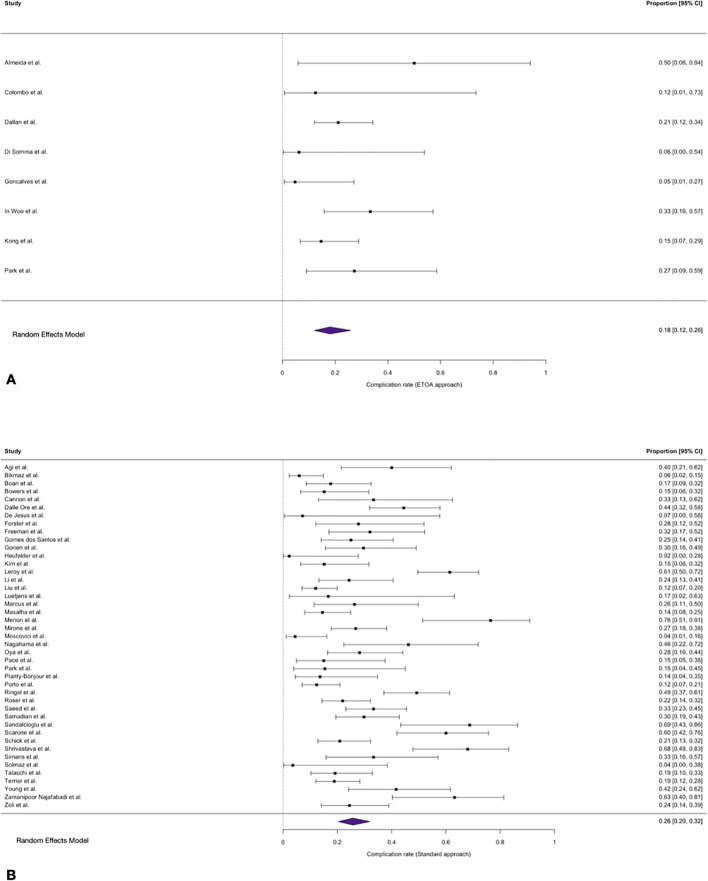
Forest plot showing the complication rate across studies included. **(A)** ETOA; **(B)** Transcranial approach.

Meta-regression did not demonstrate a significant association between surgical approach and complication rate (QM = 1.93, p = 0.165; OR = 1.86, 95% CI 0.78–4.46, trasncranial approaches vs ETOA). No influential studies were detected (**Figures 7A, B**).

Across the included studies, the qualitative profile of reported complications differed between surgical approaches. In studies reporting ETOA, the most frequent complications were predominantly orbital and cranial nerve–related, including diplopia or ocular motility disturbances (12, 35, 39, 68), ptosis (11, 23, 51), and trigeminal sensory disturbances (29, 35). No study identified major intracranial, vascular, or infectious complications as the most frequent adverse event following ETOA. In contrast, studies reporting standard transcranial approaches demonstrated a broader spectrum of complication types. While ocular motor disturbances remained the most frequently reported complication (6, 7, 22, 25, 27, 30, 34, 36, 38, 41, 43, 48–50, 53, 55, 58, 59, 61, 63–65), additional complications included trigeminal sensory deficits (3, 26, 54, 66), visual worsening (40, 42, 44), and less commonly periorbital edema (33), cerebrospinal fluid leak (60), infection (47), hygroma (56), vascular events (12), and enophthalmos (24). These findings indicate greater heterogeneity in complication type following transcranial approaches.

**Table 2** illustrates the distribution of complication types across studies. ETOA-related complications were mainly limited to orbital and cranial nerve disturbances, most frequently diplopia (4 studies) and ptosis (3 studies). In contrast, transcranial approaches showed a broader and more heterogeneous complication profile, with diplopia reported in 22 studies and a wider range of additional complications, each less consistently described.

**Table 2:** Frequency of most frequently reported complication types by surgical approach.

Complication category	Number of studies
Endoscopic Transorbital Approach (ETOA)	
Diplopia / ocular motility disturbance	4
Ptosis (including transient)	3
Trigeminal sensory disturbance (V2 hypoesthesia)	2
**Total studies reporting ETOA complications**	**9**
Standard Transcranial Approaches	
Diplopia / ophthalmoplegia	22
Trigeminal sensory disturbance	4
Visual worsening (transient or permanent)	3
Periorbital edema	1
Vascular complications (vasospasm)	1
CSF leak	1
Infection (wound infection)	1
Hygroma	1
Enophthalmos	1
Third nerve palsy (isolated reporting)	1
**Total studies reporting transcranial complications**	**36**

Bold values are the total numbers for section.

### Recurrence and progression

Follow-up duration varied substantially across the included studies. The median follow-up in studies reporting trasncranial approaches was 52 months (range 6–136 months), whereas studies employing ETOA approaches reported a median duration of 18 months (range 6–45 months).

The pooled recurrence rate following trasncranial approaches was 11% (95% CI 8-15%), with substantial between-study heterogeneity. In ETOA series, the pooled recurrence rate was 2% (95% CI 0–15%). No influential studies were identified. The pooled progression rate after trasncranial approaches was 17% (95% CI 12–24%). In ETOA series, the pooled progression rate was 4% (95% CI 1–10%), with marked between-study heterogeneity. No influential studies were identified.

**Figure 8:**
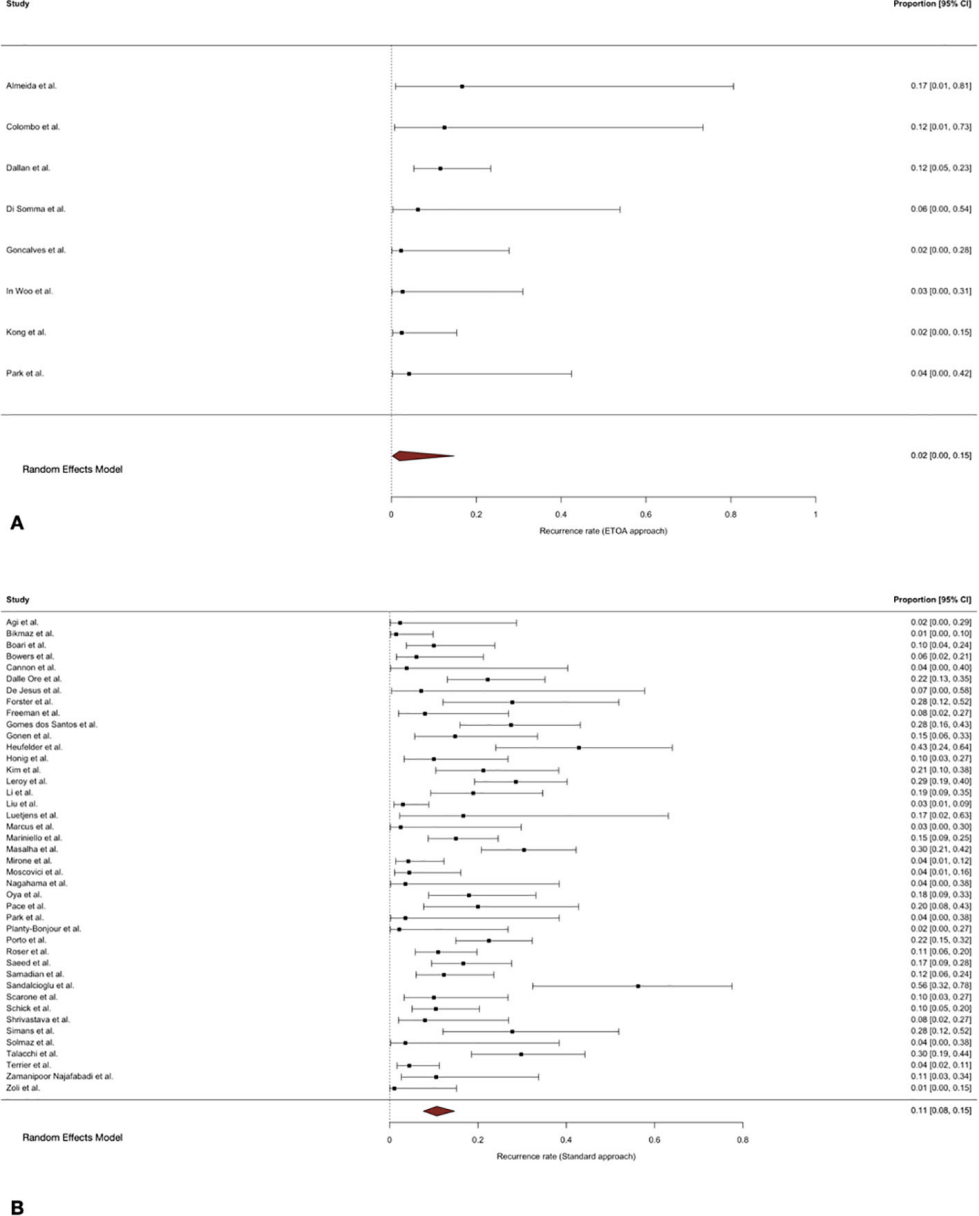
Forest plot showing the recurrence rate across studies included. **(A)** ETOA; **(B)** Transcranial approach.

**Figure 9:**
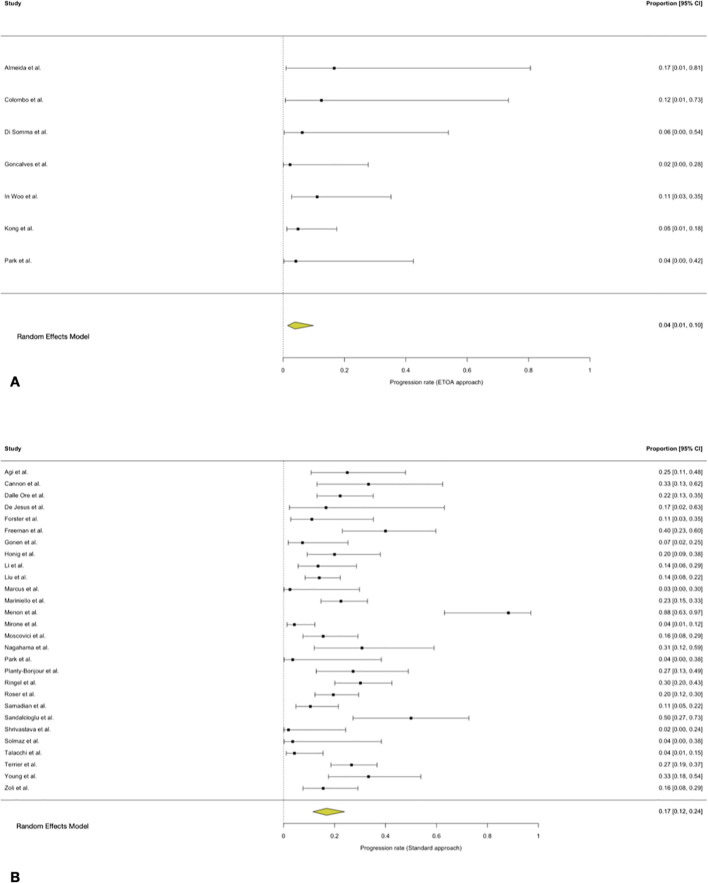
Forest plot showing the progression rate across studies included. **(A)** ETOA; **(B)** Transcranial approach.

Given the substantial imbalance and heterogeneity in follow-up duration between trasncranial and ETOA series, comparative meta-regression analyses for tumour control outcomes were not performed (**Figures 8A, B**, **9A, B**).

### Quality assessment

Using the JBI checklist for case series, 53 studies were assessed as having a low risk of bias and were therefore included (**Table 3**).

**Table 3:** Risk of bias - JBI checklist for case series.

Study	Year	1. Were there clear criteria for inclusion in the case series?	2. Was the condition measured in a standard, reliable way for all participants included in the case series?	3. Were valid methods used for identification of the condition for all participants included in the case series?	4. Did the case series have consecutive inclusion of participants?	5. Did the case series have complete inclusion of participants?	6. Was there clear reporting of the demographics of the participants in the study?	7. Was there clear reporting of clinical information of the participants?	8. Were the outcomes or follow up results of cases clearly reported?	9. Was there clear reporting of the presenting site(s)/clinic(s) demographic information?	10. Was statistical analysis appropriate?	Include
Agi et al. (22)	2020	×	✓	✓	✓	✓	✓	✓	✓	✓	✓	✓
Almeida et al. (23)	2018	✓	✓	✓	✓	✓	✓	✓	✓	✓	✓	✓
Bikmaz et al. (24)	2007	✓	✓	✓	✓	✓	✓	✓	✓	✓	✓	✓
Boari et al. (25)	2013	✓	✓	✓	✓	✓	✓	✓	✓	✓	✓	✓
Bowers et al. (26)	2016	✓	✓	✓	✓	✓	✓	✓	✓	✓	✓	✓
Cannon et al. (27)	2009	×	✓	✓	✓	✓	×	✓	✓	✓	✓	✓
Colombo et al. (28)	2022	✓	✓	✓	✓	✓	✓	✓	✓	✓	✓	✓
Dallan et al. (29)	2025	✓	✓	✓	✓	✓	×	✓	✓	✓	✓	✓
Dalle Ore et al. (30)	2020	×	✓	✓	✓	✓	×	✓	✓	×	✓	✓
De Jesus et al.	2001	✓	✓	✓	✓	✓	✓	✓	✓	✓	✓	✓
Di Somma et al. (17)	2023	✓	✓	✓	✓	✓	✓	✓	✓	✓	✓	✓
Forster et al. (33)	2014	✓	✓	✓	✓	✓	✓	✓	✓	✓	✓	✓
Freeman et al. (34)	2017	✓	✓	✓	✓	✓	✓	✓	✓	✓	×	✓
Gomes dos Santos et al.	2022	✓	✓	✓	✓	✓	✓	✓	✓	✓	✓	✓
Goncalves et al. (35)	2020	✓	✓	✓	✓	✓	✓	✓	✓	✓	✓	✓
Gonen et al. (36)	2017	×	✓	✓	✓	✓	✓	✓	✓	✓	✓	✓
Heufelder et al. (37)	2009	✓	✓	✓	✓	✓	✓	✓	✓	✓	✓	✓
Honig et al. (38)	2010	✓	✓	✓	✓	✓	✓	✓	✓	✓	✓	✓
In Woo et al. (39)	2021	✓	✓	✓	✓	✓	✓	✓	✓	✓	✓	✓
Kim et al. (40)	2022	✓	✓	✓	✓	✓	✓	✓	✓	✓	✓	✓
Kong et al.	2020	✓	✓	✓	✓	✓	✓	✓	✓	✓	✓	✓
Leroy et al. (41)	2016	✓	✓	✓	✓	✓	✓	✓	✓	✓	✓	✓
Li et al. (42)	2009	✓	✓	✓	✓	✓	✓	✓	✓	✓	✓	✓
Liu et al. (43)	2024	✓	✓	✓	✓	✓	✓	✓	✓	✓	✓	✓
Luetjens et al. (44)	2011	✓	✓	✓	✓	✓	✓	✓	✓	✓	✓	✓
Marcus et al. (45)	2013	✓	✓	✓	✓	✓	✓	✓	✓	✓	✓	✓
Mariniello et al. (46)	2024	✓	✓	✓	✓	✓	✓	✓	✓	✓	✓	✓
Masalha et al. (47)	2021	✓	✓	✓	✓	✓	✓	✓	✓	✓	×	✓
Menon et al. (48)	2020	✓	✓	✓	✓	✓	✓	✓	✓	✓	✓	✓
Mirone et al. (49)	2009	✓	✓	✓	✓	✓	✓	✓	✓	✓	✓	✓
Moscovici et al. (50)	2024	×	✓	✓	✓	✓	×	✓	✓	×	✓	✓
Nagahama et al. (51)	2019	✓	✓	✓	✓	✓	✓	✓	✓	✓	✓	✓
Oya et al. (3)	2011	✓	✓	✓	✓	✓	✓	✓	✓	✓	✓	✓
Pace et al. (52)	2019	✓	✓	✓	✓	✓	✓	✓	✓	✓	✓	✓
Park et al. (12)	2020	✓	✓	✓	✓	✓	✓	✓	✓	✓	✓	✓
Planty-Bonjour et al. (53)	2024	×	✓	✓	✓	✓	×	✓	✓	✓	✓	✓
Porto et al. (54)	2025	✓	✓	✓	✓	✓	✓	✓	✓	✓	✓	✓
Ringel et al. (55)	2006	×	✓	✓	✓	✓	×	✓	✓	×	✓	✓
Roser et al. (56)	2005	✓	✓	✓	✓	✓	✓	✓	✓	✓	✓	✓
Saeed et al. (6)	2011	✓	✓	✓	✓	✓	✓	✓	✓	✓	✓	✓
Samadian et al. (57)	2020	✓	✓	✓	✓	✓	✓	✓	✓	✓	✓	✓
Sandalcioglu et al. (58)	2005	✓	✓	✓	✓	✓	✓	✓	✓	✓	✓	✓
Scarone et al. (59)	2009	✓	✓	✓	✓	✓	✓	✓	✓	✓	✓	✓
Schick et al. (60)	2006	×	✓	✓	✓	✓	×	✓	✓	✓	✓	✓
Shrivastava et al. (7)	2005	×	✓	✓	✓	✓	×	✓	✓	×	✓	✓
Simans et al. (61)	2013	✓	✓	✓	✓	✓	✓	✓	✓	✓	✓	✓
Solmaz et al. (62)	2014	✓	✓	✓	✓	✓	✓	✓	✓	✓	✓	✓
Talacchi et al. (63)	2014	✓	✓	✓	✓	✓	✓	✓	✓	✓	✓	✓
Terrier et al. (64)	2018	✓	✓	✓	✓	✓	✓	✓	✓	✓	×	✓
Young et al. (65)	2019	✓	✓	✓	✓	✓	✓	✓	✓	✓	✓	✓
Zamanipoor Najafabadi et al. (66)	2021	✓	✓	✓	✓	✓	✓	✓	✓	✓	✓	✓
Zohdy et al. (67)	2024	✓	✓	✓	✓	✓	✓	✓	✓	✓	✓	✓
Zoli et al. (68)	2023	×	✓	✓	✓	✓	×	✓	✓	✓	✓	✓

## Discussion

This meta-analysis aimed to evaluate the comparative effectiveness and safety of ETOA and standard transcranial approaches for the management of SOMs, with a quantitative assessment of functional and anatomical outcomes (proptosis and visual function), extent of resection, and surgical morbidity, and a secondary evaluation of tumor control. This question is timely because contemporary SOM surgery has increasingly shifted toward a “function-oriented” strategy, prioritizing optic canal and orbital decompression and cosmetic and functional improvement over maximal resection when radicality carries a higher risk of approach-related morbidity or is anatomically unrealistic. Recent clinical frameworks and reviews emphasize tailoring the surgical corridor to the tumor epicenter, hyperostotic burden, and the clinical presentation (vision versus proptosis), rather than applying a single “one-size-fits-all” approach (46).

Proptosis outcomes were clearly superior with ETOA in our analysis. This aligns tightly with recent ETOA-specific systematic reviews and clinical series, which consistently report high rates of orbital decompression and proptosis improvement, reflecting the direct access of the transorbital corridor to the lateral orbital wall, sphenoid wing hyperostosis, and intraorbital tumor component (1, 8, 11, 13). Emerging volumetric/correlation work also reinforces that proptosis is strongly linked to hyperostotic and intraorbital burden, which are precisely the targets where transorbital routes can be particularly efficient (5, 29, 46). Taken together, our findings support the ETOA’s comparative advantage to achieve high-value proptosis control, through focused bony orbital work (17).

Despite different resection philosophies, visual improvement was similar and overall modest in both groups. This is not unexpected in SOM, where visual compromise may reflect chronic optic neuropathy, canalicular constriction from hyperostosis, and ischemic injury, factors that limit postoperative recovery even after technically adequate decompression (3). Contemporary series and focused discussions increasingly underscore that optic canal decompression (often with hyperostotic drilling) is the key actionable step for vision preservation or improvement, while the degree of soft intracranial tumor removal may be less predictive than timing, baseline vision, and adequacy of decompression (5, 7). In this context, the comparable visual outcomes between ETOA and transcranial approaches support the interpretation that, when the optic canal/orbit are properly addressed, approach selection alone may not be the dominant driver of visual recovery (2). Rather, visual outcomes appear to be largely driven by the preoperative severity and duration of optic nerve impairment.

In our pooled analysis, STR was more frequent with ETOA, whereas GTR favored standard transcranial approaches once influential studies were trimmed. The sensitivity analysis, in which exclusion of high-performing endoscopic series (11, 12) reinforced the advantage of transcranial approaches for GTR, underscores an important real-world consideration: endoscopic radicality is highly operator- and center-dependent, and currently reported outcomes likely reflect a combination of learning curve effects, careful case selection, and institutional experience with endoscopic skull base surgery (69). Beyond these contextual factors, there are intrinsic technical considerations that may limit the consistency of GTR with ETOA. The transorbital corridor is inherently a keyhole approach, optimized for targeted decompression and compartment-specific tumor control rather than circumferential dural clearance. Achieving radical resection may be particularly challenging when tumors extend deeply into the middle cranial fossa, cavernous sinus region, or infratemporal compartment, where critical arterio-nervous structures, including the internal carotid artery and cranial nerves within the superior orbital fissure and cavernous sinus, constrain safe dissection. In addition, management of extensive dural tails and hyperostotic bone beyond the immediate orbital and sphenoid compartments may be technically more demanding through a transorbital route compared with wider transcranial exposures. Importantly, these limitations do not negate the technical feasibility or oncological value of ETOA in selected cases but rather emphasize that maximal radicality is not uniformly achievable across all anatomical patterns using a single corridor. This observation is consistent with foundational studies that have carefully defined the indications, technical constraints, and current limitations of ETOA for SOMs, reinforcing the need for individualized, anatomy-driven surgical strategy selection (16, 17). Looking forward, an additional nuance that warrants systematic evaluation is the impact of visualization modality (endoscopy, microscopy or exoscopy) on the achievable extent of resection within transorbital corridors, as differences in illumination, depth perception, and ergonomics may further influence surgical radicality (70).

Surgical burden and tumor control require a nuanced interpretation. We found similarly low complication rates across groups, consistent with modern reports that, in experienced hands, both trasncranial approaches and ETOA can be performed with a favorable morbidity profile and careful cranial nerve preservation (11, 71). We observed low complication rates with both approaches; however, complications were numerically more frequent following standard transcranial surgery than after ETOA, although this difference did not reach statistical significance. Importantly, the type of complications differed between approaches: ETOA was mainly associated with orbital and cranial nerve–related events, whereas transcranial approaches showed a broader spectrum of approach-related morbidity, including visual deterioration, cerebrospinal fluid leak, vascular, and infectious complications. This is consistent with modern reports demonstrating that, in experienced hands, both trasncranial approaches and ETOA can be performed with a favorable morbidity profile and careful cranial nerve preservation (8, 65). Recurrence and progression appeared low in both cohorts and numerically lower after ETOA; however, our results are in line with updated reviews that repeatedly warn that ETOA follow-up is often shorter, which can artifactually deflate recurrence and progression estimates compared with transcranial series (1, 13). The slightly higher progression rate observed in standard approaches may therefore reflect longer surveillance rather than inferior surgery, especially given the known long natural history of residual SOM and the frequent use of staged or adjuvant strategies (4, 7). Taken together, these considerations underscore the importance of long-term, continuative follow-up in the management of these patients to accurately assess tumor control and guide timely adjunctive interventions.

Overall, our synthesis supports a balanced interpretation of the current evidence. ETOA represents a versatile and increasingly established surgical corridor for SOMs, offering effective access to the orbit, anterior cranial fossa, middle cranial fossa, and infratemporal region, which correspond to the typical patterns of tumor extension. In many cases, this access enables satisfactory orbital decompression, optic canal management, and clinically meaningful tumor resection with favorable functional outcomes. At the same time, standard transcranial approaches continue to play an important role, particularly in the presence of extensive intracranial or dural disease, where wider exposure may facilitate more aggressive dural control and potentially higher rates of gross total resection. Rather than positioning one strategy as superior, these findings support a tailored, anatomy- and goal-driven approach, in which ETOA and transcranial techniques are complementary tools within the surgical armamentarium. Ongoing maturation of long-term outcome data will be essential to further clarify their relative roles in durable tumor control (2, 3). Finally, the high heterogeneity observed across pooled outcomes in this study further underscores the intrinsic complexity and heterogeneity of SOM management, reflecting variability in tumor biology, patterns of osseous and dural involvement, baseline visual status, and surgical intent.

### Strengths and limitations

This study represents, to our knowledge, the first meta-analysis specifically designed to evaluate the influence of surgical approach on outcomes in SOMs, analyzing ETOA and standard transcranial techniques. The quantitative synthesis includes more than 2,000 patients drawn from 53 studies, providing the largest quantitative synthesis currently available for this disease entity. The inclusion of experiences from high-volume referral centers as well as smaller institutions enhances the external validity of the findings and reflects real-world heterogeneity in surgical practice. In addition, the use of random-effects generalized linear mixed models, approach-specific meta-regression, and structured influence and sensitivity analyses strengthens the robustness of the results and allows a nuanced interpretation of the impact of surgical approach across heterogeneous study designs.

Several limitations must be acknowledged. First, the available literature is largely composed of retrospective, non-comparative case series, with a paucity of direct head-to-head comparative studies between ETOA and standard transcranial approaches, limiting causal inference. Second, the ETOA cohort represents a relatively small proportion of the overall sample, reflecting the recent adoption of this technique and potentially reducing statistical power for some outcomes. Third, follow-up duration was substantially shorter in ETOA series compared with transcranial cohorts, precluding meaningful comparative meta-regression for tumor control outcomes and likely underestimating recurrence and progression rates after ETOA. Fourth, operative time was inconsistently reported and variably defined across studies, preventing reliable comparison between approaches. This limitation is particularly relevant given that operative duration may reflect surgical complexity, learning curve effects, resource utilization, and potentially perioperative morbidity. The inability to account for operative time, therefore limits interpretation of procedural efficiency and may obscure differences related to surgical experience rather than intrinsic approach-related factors. Finally, heterogeneity in patient selection, surgical goals, extent of hyperostosis resection, reconstruction strategies, and outcome definitions across studies may have contributed to the high between-study variability observed in several analyses.

## Conclusions

This meta-analysis provides a comparative synthesis of ETOA and standard transcranial approaches for the management of SOMs, highlighting the strengths and limitations of each strategy. While traditional transcranial approaches continue to offer higher rates of GTR and durable intracranial and dural control, ETOA demonstrates comparable safety and visual outcomes, with a clear advantage in the management of proptosis through targeted orbital and hyperostotic bone decompression.

These findings support a contemporary, patient-centered and function-oriented surgical paradigm in which ETOA is considered an anatomically tailored and goal-directed option within the surgical armamentarium for SOMs, particularly in selected cases with predominant orbital involvement, while transcranial approaches continue to be a workhorse for SOM management depending on tumor extent and surgical objectives. Given the relative novelty of transorbital approaches and the shorter follow-up available in current series, longer-term and multicenter data are required to better define their oncologic durability and reproducibility.

## Data Availability

The original contributions presented in the study are included in the article/supplementary material, further inquiries can be directed to the corresponding author/s.
